# Racial Implicit Bias and Communication Among Physicians in a Simulated Environment

**DOI:** 10.1001/jamanetworkopen.2024.2181

**Published:** 2024-03-20

**Authors:** Cristina M. Gonzalez, Tavinder K. Ark, Marla R. Fisher, Paul R. Marantz, Diana J. Burgess, Felise Milan, Malika T. Samuel, Monica L. Lypson, Carlos J. Rodriguez, Adina L. Kalet

**Affiliations:** 1 Institute for Excellence in Health Equity, New York University Grossman School of Medicine, New York; 2 Department of Medicine, New York University Grossman School of Medicine, New York; 3 Department of Population Health, New York University Grossman School of Medicine, New York; 4 Medical College of Wisconsin, Milwaukee; 5 Department of Psychiatry, Mount Sinai Morningside-West, New York, New York; 6 Department of Epidemiology and Population Health, Albert Einstein College of Medicine, Bronx, New York; 7 Department of Medicine, Albert Einstein College of Medicine, Bronx, New York; 8 Department of Medicine, University of Minnesota, Minneapolis; 9 Center for Care Delivery and Outcomes Research in the Minneapolis Veterans Affairs Healthcare System, Minneapolis, Minnesota; 10 Department of Medicine, Albert Einstein College of Medicine/Montefiore Medical Center, Bronx, New York; 11 New York University Tisch School of the Arts, New York; 12 Columbia University Vagelos College of Physicians and Surgeons, New York, New York; 13 Department of Medicine, Columbia University Irving Medical Center, New York, New York; 14 Department of Medicine and Epidemiology & Population Health, Albert Einstein College of Medicine/Montefiore Medical Center, Bronx, New York

## Abstract

**Importance:**

Racial implicit bias can contribute to health disparities through its negative influence on physician communication with Black patients. Interventions for physicians to address racial implicit bias in their clinical encounters are limited by a lack of high-fidelity (realistic) simulations to provide opportunities for skill development and practice.

**Objective:**

To describe the development and initial evaluation of a high-fidelity simulation of conditions under which physicians might be influenced by implicit racial bias.

**Design, Setting, and Participants:**

This cross-sectional study, performed on an online platform from March 1 to September 30, 2022, recruited a convenience sample of physician volunteers to pilot an educational simulation.

**Exposures:**

In the simulation exercise, physicians saw a 52-year-old male standardized patient (SP) (presenting as Black or White) seeking urgent care for epigastric pain, nausea, and vomiting. The case included cognitive stressors common to clinical environments, including clinical ambiguity, stress, time constraints, and interruptions. Physicians explained their diagnosis and treatment plan to the SP, wrote an assessment and management plan, completed surveys, and took the Race Implicit Association Test (IAT) and Race Medical Cooperativeness IAT. The SPs, blinded to the purpose of the study, assessed each physician’s communication using skills checklists and global rating scales.

**Main Outcomes and Measures:**

Association between physicians’ IAT scores and SP race with SP ratings of communication skills.

**Results:**

In 60 physicians (23 [38.3%] Asian, 4 [6.7%] Black, 23 [38.3%] White, and 10 [16.7%] other, including Latina/o/x, Middle Eastern, and multiracial; 31 [51.7%] female, 27 [45.0%] male, and 2 [3.3%] other), the interaction of physicians’ Race IAT score and SP race was significant for overall communication (mean [SD] β = −1.29 [0.41]), all subdomains of communication (mean [SD] β = −1.17 [0.52] to −1.43 [0.59]), and overall global ratings (mean [SD] β = −1.09 [0.39]). Black SPs rated physicians lower on communication skills for a given pro-White Race IAT score than White SPs; White SP ratings increased as physicians’ pro-White bias increased.

**Conclusions and Relevance:**

In this cross-sectional study, a high-fidelity simulation calibrated with cognitive stressors common to clinical environments elicited the expected influence of racial implicit bias on physicians’ communication skills. The outlined process and preliminary results can inform the development and evaluation of interventions that seek to address racial implicit bias in clinical encounters and improve physician communication with Black patients.

## Key Points

**Question:**

Can standardized patients in a simulated environment be effectively used to explore racial implicit bias and communication skills among physicians?

**Findings:**

In this cross-sectional study of 60 physicians, a simulated environment calibrated with cognitive stressors common to clinical environments resulted in expected communication patterns based on prior research (performed in actual clinical environments) on racial implicit bias and physician communication. Higher physician pro-White implicit bias was associated with more positive ratings by White standardized patients on communication skills; conversely, Black standardized patients rated physicians more negatively.

**Meaning:**

This simulation and the process of its development can inform interventions that provide opportunities for skills development and assessment of skills in addressing racial implicit bias.

## Teaser

This cross-sectional study of physician volunteers describes a high-fidelity simulation that included cognitive stressors common to clinical environments designed to precipitate conditions under which physicians might be influenced by their implicit racial bias.

## Introduction

Racial implicit bias negatively influences physician communication with Black patients.^1,2,3,4^ It is commonly measured using the Race Implicit Association Test (IAT), a validated online latency response test that measures reaction times to matching images (eg, faces of Black and White adults) and value-laden words (eg, joy and evil).^5,6^ Physicians with higher Race IAT scores indicating more pro-White bias demonstrate more verbal dominance,^1^ lower patient affect scores,^1^ less supportive communication,^2^ shorter interactions,^2^ and increased use of words that reflect social dominance and anxiety^3^ when caring for Black compared with White patients. Higher physician racial implicit bias is associated with lower perceived patient centeredness^1,2,4^ and greater difficulty remembering contents of the conversation after an encounter with a physician^3^ for Black compared with White patients. Poor communication outcomes have downstream health effects, including delays or avoidance in seeking medical care and decreased patient adherence to treatment plans.^7^

Given the negative influence of racial implicit bias on physician communication with Black patients^1,2,3,4^ and the contributions of implicit bias in general to health care disparities,^8^ addressing implicit bias has become a focus of medical education.^9,10,11,12,13,14,15,16,17,18,19,20,21,22^ Addressing implicit bias is mandated by accreditation bodies for undergraduate and graduate medical education.^23,24^ Training in addressing implicit bias has been suggested for all practicing physicians.^25^ To date, most interventions focus on raising self-awareness of implicit bias^10,11,26,27,28,29,30,31^; although important and necessary, awareness is not sufficient. A careful review of the literature shows that raising self-awareness without providing skills training has no demonstrated efficacy for improving behaviors^32,33^ and is associated with negative outcomes and unintended consequences, such as avoidant behaviors.^34^

To address the limitations of prior approaches to addressing implicit bias, our group developed a skills-based, behavioral approach to addressing implicit bias within clinical encounters.^16,35^ Such observable skills and behaviors could be assessed in clinical encounters with Black and White patients, allowing for the evaluation of interventions seeking to enhance a physician’s ability to address implicit bias. To date, no tools exist to assess the effect of any skills-based interventions on physician communication skills. Given unintended consequences of other implicit bias approaches,^34^ it is crucial to pilot skills-based interventions and assessments using simulations to avoid unintentionally causing harm to Black patients.

Few simulations focus on physician implicit bias and its impact on physician communication skills,^36^ and none to our knowledge quantify the association between implicit bias and communication skills. Clinical environments are often replete with cognitive stressors, including clinical ambiguity, stress, time constraints, and interruptions; implicit bias is more likely to negatively impact a clinical encounter in the setting of these cognitive stressors.^37^ Without high-fidelity (ie, realistic) simulations that include cognitive stressors common to clinical environments, we will be unable to advance opportunities for physicians to practice skills in addressing implicit bias that could eventually improve patient-physician communication. Moreover, we will remain unable to evaluate the efficacy of any skills-based interventions to address the negative effect of implicit bias without exposing patients to unintended consequences if we do not develop high-fidelity simulations. To address this important gap, we developed a high-fidelity simulation that included cognitive stressors common to clinical environments designed to precipitate conditions under which physicians might be influenced by their implicit bias. We describe the development of this simulation, detail the associated communication skills assessments, and present the results of our initial proof-of-concept pilot study.

## Methods

### Scenario Development

This cross-sectional study, conducted from March 1 to September 30, 2022, recruited a convenience sample of physician volunteers to pilot an educational simulation. Due to the COVID-19 pandemic, we conducted all procedures via an online platform (Zoom, Zoom Video Communications Inc). We iteratively developed a 3-station simulation that consisted of 3 standardized patient (SP) cases. We piloted and debriefed cases with SPs, trained observers serving as monitors, the investigative team, and local physician volunteers to identify components of the simulation needing revision. The SPs were actors who were unaware of the purpose of the simulation and only gave feedback on aspects specific to the script (nothing about racial implicit bias). Monitors, who were also actors, were aware of the simulation’s purpose and were trained to observe cases and give feedback from that vantage point. Local physician volunteers (N = 28) completed the simulations and all procedures described in the pilot study, gave feedback to the investigators on improving the fidelity of the cases, advised on technical glitches, and highlighted inconsistencies in the questionnaires. The first case in this 3-station simulation exercise (practice scenario) was a young woman reticent to give her history. This case was unscored to minimize construct-irrelevant variance in subsequent scenarios that might result from unfamiliarity with interacting with the SP on the online platform. The second case (study scenario), the unit of analysis for this investigation, was a man with acute coronary syndrome (ACS). The third case (not discussed in this article) involved a woman presenting with acute onset of pain under her right breast. We also sought community member input by conducting four 60-minute focus groups with a total of 17 community members to obtain feedback on the realistic nature of the simulation from the patient perspective; community members received a $50 gift card to participate (C.M.G., unpublished data, April 29, 2021). This study was approved by the institutional review board of the Albert Einstein College of Medicine. All participants provided written informed consent. This study followed the Strengthening the Reporting of Observational Studies in Epidemiology (STROBE) reporting guideline.^38^

#### Study Scenario

Acute coronary syndrome, a clinical situation with known health care disparities, was chosen for the study scenario.^39,40,41,42,43^ In collaboration with a cardiologist (C.J.R.), we developed a case of a 52-year-old man with ACS who presented with epigastric pain that had increased in frequency and intensity during the past few months, with onset of nausea and vomiting since the night before presentation. The patient, asymptomatic at the time of presentation, was mildly diaphoretic on physical examination; electrocardiography revealed only left ventricular hypertrophy (thickened heart muscle) but no acute findings. The SP was trained to provide all the correct information when asked but only admitted to the progressively worsening course of symptoms if specifically asked. This symptom complex was not the crushing, substernal chest pain typical of ACS and was therefore clinically ambiguous, creating a diagnostic challenge for physicians, serving as a cognitive stressor and increasing cognitive load.

#### Cognitive Stressors

Scenarios included cognitive stressors common to clinical environments.^37,44,45^ The practice scenario that preceded the study scenario was an emotionally charged encounter intended to result in some cognitive depletion for the physicians.^37^ All SPs were pleasant but somewhat meandering in their responses, answering specific questions about their symptoms if directly asked but not offering information freely. Physicians were given 15 minutes per case, with a time-call interruption at 5 minutes remaining. In the study scenario, a monitor playing a standardized nurse interrupted the encounter at 9 minutes remaining and informed the participant that their next patient had arrived and was in a lot of pain. Toward the end of the study scenario, the SPs requested their wives be called because they did not understand the diagnosis and treatment plan offered by the physician.

#### Selection and Training of SPs and Monitors

In collaboration with SP educators, we cast both Black and White men to play our study scenario patient, Mr Richard Grant. All men had prior experience as SPs. We selected SPs with similar English fluency and body mass index; they wore similar clothing and had a standard virtual backdrop. Following published guidelines for planning and implementing SP simulations,^46,47^ we conducted extensive training (mean, 11 hours; range, 4-22 hours) to standardize performance and frame of reference rater training (interrater training) to create a shared understanding of the skills checklists and global rating scales to standardize the assessment of physician performance (mean, 4.5; range, 2-10 hours).^48,49^ The SPs remained blinded to the purpose of the study throughout. We ran 2 simulations at a time in separate breakout rooms over Zoom, with one staffed by a Black SP and the other by a White SP.

Conducting the simulation on an online platform required training of monitors to keep time, make announcements, read the narrated physical examination, and interrupt the encounter. Monitors only came on camera to narrate the physical examination and to play the standardized nurse who enters the room to interrupt the encounter. The monitors were not selected for race or other demographic factors. They also received extensive training to ensure a standardized experience across breakout rooms (mean training time, 9 hours; range, 3-28 hours).

#### Data Collection and Outcome Measures: SP Skills Checklists and Global Rating Scales

We developed the skills checklist by adapting an existing 12-item interpersonal communication skills scale based on the 3-function model of medical interviewing: (1) build the relationship, (2) assess and understand, and (3) collaborative management.^50^ The adaptations made included verbal and nonverbal behaviors perceived as biased by patients that had been correlated with physician implicit bias in actual clinical encounters (eg, patient-physician rapport items).^1,2,3,4^ We did not add items that assessed safe touch, attention to patient’s physical comfort, or interpersonal distance due to the need to conduct this simulation on an online platform.^1,2,4,51,52,53^ The SPs completed skills checklists (including communication skills subdomains of information gathering, listening, relationship development, patient education, and rapport) and global rating scales at the end of each encounter. In response to SP feedback, during the development phase, we extended the original 3-point scale to a 5-point checklist item response scale, with 1 indicating not done and 5 indicating well done (eAppendix in Supplement 1).

### Pilot Study

#### Participants and Setting

We recruited physician volunteers in either residency or within their first 5 years on faculty in internal medicine or family medicine across institutions in New York City. Physicians were told only that they were participating in piloting educational simulations. Racial bias was not mentioned. Data on race and gender were self-reported within the demographic data survey (Table 1). Key stakeholders, such as program directors or division chiefs, at major academic medical centers in New York City sent email invitations. Potential participants scanned a QR code and were screened for eligibility and, if eligible, signed up for a specified time on 1 of 7 simulation days. We focused on early career physicians to keep experience levels somewhat similar across participants as we calibrated cognitive stressors (including clinical ambiguity).

**Table 1.** Demographic Summary and Mean Race and Race and Medical Cooperativeness IAT Scores for Physicians in a High-Fidelity Simulation Study

Characteristic	No. (%) of physicians	Mean (SD) scores^a^	
		Race IAT	Race and Medical Cooperativeness IAT
Race			
Asian	23 (38.3)	0.446 (0.388)	0.194 (0.414)
Black	4 (6.7)	−0.070 (0.450)	−0.362 (0.251)
White	23 (38.3)	0.419 (0.432)	0.148 (0.361)
Other^b^	10 (16.7)	0.434 (0.387)	0.158 (0.245)
Gender			
Female	31 (51.7)	0.287 (0.431)	0.080 (0.316)
Male	27 (45.0)	0.565 (0.356)	0.191 (0.450)
Other^c^	2 (3.33)	0.035 (0.317)	0.301 (0.038)

Abbreviation: IAT, Implicit Association Test.

^a^ IAT scores range from −2.0 to 2.0. Scores above 0.35 and 0.65 indicate a moderate and strong pro-White bias, respectively.

^b^ Other races were Latina/o/x, Middle Eastern, and multiracial.

^c^ Other gender indicates prefer not to say.

#### Procedure

When logging into the Zoom platform, physicians were assigned a randomly generated 4-digit identification code, received a brief orientation to the overall session, and had their questions answered. Physicians then completed a brief survey of demographic data (Table 1). At the beginning of each encounter, physicians were given a “door note” with the patient’s name, chief concern, vital signs, and an advisement that all patients underwent electrocardiography and chest radiography on triage at the urgent care center. Physicians obtained a history, received a narrated physical examination and radiographic results, and reviewed the electrocardiogram. They then discussed their diagnostic and treatment plans with the SP. Using Qualtrics surveys after each encounter, physicians answered questions regarding their perceptions of the simulation, and SPs completed the communication skills checklists. Physicians’ final task after the third station was to complete the Race IAT, which was chosen due to evidence suggesting scores are associated with behaviors toward Black individuals.^6,54^ The Race and Medical Cooperativeness IAT measures mental associations between race and cooperativeness with medical recommendations (eAppendix 2 in Supplement 1)^1^; this second IAT was custom-made for this study following the format used by Cooper et al.^1^ All physicians received a $100 gift card in appreciation of their time.

The principal investigator (C.M.G.) conducted a debrief with participants whose purpose was 2-fold: to reveal the full purpose of the study, addressing any questions or reactions that arose, and to obtain participant feedback. For the latter, participants were invited to discuss their perceptions of each procedure within the study. They were asked if they had taken an IAT before and if they knew about the full purpose of the study before participating. The principal investigator (C.M.G.) took notes, which were reviewed by the investigative team to identify lessons learned.

### Statistical Analysis

Results from both IATs are reported as D scores ranging from −2.0 to 2.0, with the highgest scores indicating an extreme pro-White bias, and treated as a continuous variable in our analyses. For interpretation purposes, scores are grouped into 7 categories.^55^ For example, for the Race IAT, negative D scores are categorized strong, moderate, or slight preference for African Americans (pro-Black) as they increase from −2 toward 0. The neutral option of “little to no preference” is reserved for D scores approaching 0. As D scores become more positive, they are similarly categorized as slight, moderate, and strong preferences for European Americans (pro-White).^55^

Linear regression was conducted on the overall SP communication ratings and each subdomain, with the SP race, each of the IAT scores, and all interactions included as variables. The skills checklist data were treated as continuous in this analysis because all options on the 5-point scale were used and normally distributed, with skewness values ranging from −0.1 to 0.4. A 2-sided *P* < .05 was considered statistically significant. Analyses were conducted using R, version 4.3.2 (R Project for Statistical Computing).

## Results

We recruited 64 physicians, each seeing 1 of 9 SPs (3 Black and 6 White). Of these physicians, 29 (45.3%) were rated by a Black SP and 35 (54.7%) by a White SP, but due to missing IAT scores, the number of physicians in the analyses was 60 (23 [38.3%] Asian, 4 [6.7%] Black, 23 [38.3%] White, and 10 [16.7%] other, including Latina/o/x, Middle Eastern, and multiracial; 31 [51.7%] female, 27 [45.0%] male, and 2 [3.3%] other) for the communication analyses. Table 1 provides the mean Race IAT and Race and Medical Cooperativeness IAT scores listed by gender and race of participants. In general, participants who identified as Asian, White, or other had a moderate pro-White bias, and Black participants had a slight pro-Black bias. Male participants had a higher pro-White bias than female participants. The Race and Medical Cooperativeness IAT was offered after the Race IAT and was completed less frequently.

During the debrief, 34 participants reported that they had taken an IAT for other educational purposes. No participant knew the full purpose of the study before participating. Only 1 participant was concerned about what the IAT told the investigative team about him. We were able to reassure him that we do not use any IAT scores as a diagnostic tool about individuals but rather were looking at data across the population of participants. Participants evaluated the simulation on a 1- to 10-point scale, with 1 indicating not realistic (or not similar to clinical experiences they have experienced in their career) and 10 indicating extremely realistic (or extremely similar to clinical experiences they have experienced in their career). Fifty-two participants (86.7%) deemed the simulation realistic (rated >7/10), and 38 (63.3%) deemed the simulation similar to clinical scenarios they have experienced in their career (rated >7/10).

Table 2 provides a summary of the linear regression models for each domain measured on the skills checklists and global rating scales. The interaction of physicians’ Race IAT score and SP race was significant for overall communication (mean [SD] β = −1.29 [0.41]), all subdomains of communication (mean [SD] β = −1.17 [0.52] to −1.43 [0.59]), and overall global ratings (mean [SD] β = −1.09 [0.39]). Black SPs gave participants higher ratings than White SPs. For every unit increase in participants’ Race IAT score (suggestive of a preference for White compared with Black people), SP ratings of participant performance increased. Therefore, we included both SP race and participant Race IAT scores as an interaction term. In contrast to the positive association when each variable was analyzed independently, there was a negative association between the interaction of SP race and participants’ Race IAT scores and overall communication, all subdomains of communication, and overall global ratings. Therefore, Black SPs rated participants lower on communication skills for a given pro-White Race IAT score than White SPs. White SP ratings increased as participants’ pro-White bias increased; Black SPs rated participants with pro-White bias lower in all measures of communication skills than White SPs. In contrast, the only significant association with the Medical Cooperativeness IAT scores was with the information-gathering domain of communication skills. To preserve statistical power in this small sample, this interaction term was not included in the analyses.

**Table 2.** Summary of Linear Regression Models for Overall Communication, Each Communication Subdomain for the Skills Checklist, and Overall Global Ratings in a High-Fidelity Simulation Study

Model	Mean (SD) β						
	Overall communication	Information gathering	Listening	Relationship development	Patient education	Rapport	Overall global ratings
SP Black race	1.12 (0.25)^a^	1.01 (0.27)^a^	1.34 (0.35)^a^	1.17 (0.28)^a^	0.70 (0.32)^b^	1.34 (0.31)^a^	1.24 (0.24)^a^
Race IAT	1.31 (0.33)^a^	1.18 (0.35)^c^	1.32 (0.47)^c^	1.44 (0.37)^a^	1.42 (0.43)^c^	1.13 (0.42)^c^	1.01 (0.31)
Race Medical Cooperativeness IAT	−0.54 (0.28)	−0.73 (0.31)^b^	−0.44 (0.41)	−0.47 (0.32)	−0.63 (0.37)	−0.47 (0.36)	−0.44 (0.27)^c^
SP Black race × race IAT	−1.29 (0.41)^c^	−1.18 (0.45)^b^	−1.43 (0.59)^b^	−1.40 (0.47)^c^	−1.23 (0.54)^b^	−1.17 (0.52)^b^	−1.09 (0.39)^c^

Abbreviations: IAT, Implicit Association Test; SP, standardized patient.

^a^ *P* < .001.

^b^ *P* < .05.

^c^ *P* < .01.

## Discussion

Despite generally giving higher communication scores, Black SP ratings of participants’ communication skills across subdomains became more negative compared with White SP ratings as participants’ Race IAT scores indicated more pro-White bias. These associations are consistent with extant literature on physician communication and racial implicit bias.^1,2,3,4^ To our knowledge, although simulations incorporating implicit bias have been developed for genetic counselors,^56^ nurses,^57^ and medical residents and nurse practitioners,^36^ no published studies have measured the association of racial implicit bias with physician communication skills from the perspectives of blinded SPs, who would approximate the perspective of patients in actual clinical settings. Additionally, although prior simulation studies incorporating implicit bias intentionally selected SP identity factors, such as race or gender identity, none reported integration of cognitive stressors common to clinical environments.^36,56,57^ In this pilot study, we created a simulation that precipitates the negative influence of racial implicit bias on physician communication skills: features potentially contributing to its success were the realistic nature of the simulation and inclusion of cognitive stressors common to clinical environments.^37,44,45^

Participants deemed the simulation realistic and were able to complete all procedures online. The intentional deception we undertook by not telling participants about the racial implicit bias component of the study was necessary so that physicians would participate with as close to their natural clinical practice behaviors as possible; it has been undertaken in various studies exploring physician implicit bias.^58,59,60,61^ Previous studies reported negative reactions to implicit bias being a part of simulations,^36,57^ which highlights the importance of debriefing with a content expert, especially after intentional deception is used. The IAT is resistant to faking^62^ and is therefore well suited for the end of the simulation, so as to not prime participants to the purpose of the study. Numerous studies have shown the IAT to have strong psychometric properties, including test-retest reliability, internal consistency, and predictive validity.^54,63^

### Limitations

Our study has several limitations. The COVID-19 pandemic required that we transition to an online platform; we could not investigate nonverbal communication behaviors, such as interpersonal distance and safe touch. Additionally, the study may be underpowered to look at the influence of the Race and Medical Cooperativeness IAT because it did not have a significant association with communication scores. Prior data demonstrate that tailored IATs may yield slower responses by participants than the traditional Race IAT.^64^ Although we recruited physicians from across New York City, participants did grow up in various regions of the US and other countries; the geographic influence on perceptions and experience of caring for patients may differ by region and country. Given the relatively small sample size of this pilot study, we were not able to account for participants’ demographic variables, such as race. We will continue to investigate the impact of participants’ characteristics as we expand our study going forward.

The structure and procedure of the simulation are similar to those generally used in simulated patient encounters and Objective Structured Clinical Examinations.^46^ The similarities of our simulation to existing educational Objective Structured Clinical Examination programs, given our 3-station model, may increase uptake and opportunities for skill development and practice in addressing implicit bias for physician trainees and practicing physicians (through continuing medical education). This increase in uptake and opportunities could address some of the limitations of current approaches to addressing implicit bias. In addition, this increase may contribute to advancing the efforts of health care organizations and academic medical centers heeding the call to focus on eliminating discriminatory behaviors that result from implicit bias.^65^

## Conclusions

We created a high-fidelity simulation calibrated with cognitive stressors common to the clinical environment that elicited the expected influence of racial implicit bias on physician volunteers’ communication skills. This simulation can inform efforts to develop interventions providing opportunities for skill development and practice in addressing implicit bias for physicians and other clinicians. It can also inform the development of simulations to test the efficacy of such interventions on communication skills. Our next steps include investigating the association between diagnostic decision-making in this simulation with scores on both IATs and incorporating this simulation into an efficacy study of a novel, skills-based faculty development program.

## Notes

Supplement 1. **eAppendix 1.** Communication Skills Checklist and Global Rating Scales **eAppendix 2.** Components of Implicit Association Tests

Supplement 2.
Data Sharing Statement
