# Supplementary material for: Racial Implicit Bias and Communication Among Physicians in a Simulated Environment
Source: JAMA Netw Open. 2024 Mar 20;7(3):e242181. doi: 10.1001/jamanetworkopen.2024.2181 (PMC10955368; doi:10.1001/jamanetworkopen.2024.2181)
Supplement: Supplement 1. — eAppendix 1. Communication Skills Checklist and Global Rating Scales eAppendix 2. Components of Implicit Association Tests [file jamanetwopen-e242181-s001.pdf]

Supplementary Online Content

Gonzalez CM, Ark T, Fisher MR, et al. Assessment of racial implicit bias and communication among physicians in a simulated environment. *JAMA Netw Open*. 2024;7(3):e242181. doi:10.1001/jamanetworkopen.2024.2181

eAppendix 1. Communication Skills Checklist and Global Rating Scales

eAppendix 2. Components of Implicit Association Tests

This supplementary material has been provided by the authors to give readers additional information about their work.

© 2024 Gonzalez CM et al. *JAMA Network Open.*

eAppendix 1. Communication Skills Checklist and Global Rating Scales

Communication Skills Checklist

<u>INFORMATION GATHERING</u>

Allowing the patient to tell their story

○ 5-Well done: Asked questions one at a time without leading. Encourages and lets patient tell story with open ended questions and doesn't interrupt patient or introduce new information.

○ 4

○ 3- Partly done: Asked leading questions OR more than one question at a time. Sometimes interrupted you.

○ 2

○ 1- Not done: Asked leading questions AND more than one question at a time. Frequently interrupted you.

Elicits all patient's concerns

○ 5- Well done: Patient's concerns and understanding of problems and expectations are fully identified; specifically, physician questions for hidden concerns.

○ 4

○ 3- Partly done: Identifies some of patient's concerns but doesn't follow through with addressing concerns or identifying hidden concerns; provides some support for the patient.

○ 2

○ 1- Not done: Fails to identify/discuss patient's concerns or provide support to express emotions.

<u>FACILITATION SKILLS – Active Listening</u>

Non-verbal skills (As if you were watching the encounter on MUTE)

○ 5- Well done: Skillful use of gestures that facilitate communication. (Maintains eye contact at comfortable intervals throughout the interview, head nodding, body positioning,

© 2024 Gonzalez CM et al. *JAMA Network Open.*

physical contact, facial expression conveys interest, attentiveness and acceptance throughout the interview.)

○ 4

○ 3- Partly done: Limited use of gestures that facilitate communication. (Eye contact was made but not optimal. Body positioning, facial expression make interviewer appear somewhat interested and attentive.)

○ 2

○ 1- Not done: Lack of gestures that facilitate communication. (Does not maintain eye contact, body positioning is closed with arms folded and/or leaning back, facial expression conveys lack of interest or attentiveness, etc.)

Verbal skills (As if you were listening with your eyes closed)

○ 5- Well done: Encourages patient to speak using echoing (repeats some portion of what patient just said), "uh-huh"s, encouraging remarks, always allows sufficient pauses. If interruption is present it is skillful and appropriate. Volume of speech is ideal, facilitates communication. Tone of voice conveys interest, attentiveness and acceptance throughout the interview

○ 4

○ 3- Partly done: Some of the time encourages patient to speak. Sometimes allows sufficient pauses. Interrupts patient inappropriately some of the time. Volume of speech is not ideal, partially interferes with communication. Tone of voice made interviewer appear somewhat interested and attentive

○ 2

○ 1- Not done: Does not encourage the patient to speak (lack of encouraging remarks, "uh-huh"s or echoing). Does not allow sufficient pauses. Interrupts patient often and

© 2024 Gonzalez CM et al. *JAMA Network Open.*

unnecessarily. Volume of speech is too loud or too low, interferes with communication. Tone of voice conveys lack of interest or attentiveness.

<u>RELATIONSHIP SKILLS</u>

Respects patient

○ 5- Well done: Never condescending or judgmental in verbal or non verbal communication or tone of voice. Regards pt. as a partner, and understands pt's perspective and preferences in their healthcare. (Acknowledges phone call interruption if present.)

○ 4

○ 3- Partly done: Sometimes condescending or judgmental in verbal or non-verbal communication as well as tone of voice. Seeks pt's views about their healthcare decisions some of the time and occasionally regards patient as a partner in that process.

○ 2

○ 1- Not done: Condescending or judgmental in verbal or non-verbal communication, or tone of voice. Does not seek pt's views about their healthcare decisions. Seems rude or abrupt.

Acknowledges and legitimizes feelings, discomfort and cues

○ 5- Well done: Acknowledges pt's emotional and physical pain or discomfort. Assures pt. that they will do everything to help ease that pain. Offers words of comfort and gestures that convey sincere concern. (i.e., reflective statements, offering of pain meds.) Picks up on non-verbal cues and tries to foster a trusting environment by addressing pt fears and concerns. Never dismisses pt's feelings.

○ 4

○ 3- Partly done: Some acknowledgement of pt's emotional or physical pain and discomfort. Some use of reflective statements such as, "I see you are in a lot of pain and I

© 2024 Gonzalez CM et al. *JAMA Network Open.*

will examine you as quickly and gently as possible." However statements are not always felt to be sincere or accurate. Sometimes offers additional support.

○ 2

○ 1- Not done: No acknowledgement or legitimization of pt's emotional, physical pain or discomfort. Dismisses or diminishes pt's pain or discomfort. Oblivious to the vulnerability of the pt.

Understanding the context of the illness in the patient's life

○ 5- Well done: The interviewer inquires about the patient's feelings about their illness, how it has changed their life and tries to counsel the patient and/or recommend resources to help.

○ 4

○ 3- Partly done: The interviewer partially addresses the impact of the illness on the patient's life or self-image. And/Or The interviewer offers no counseling or resources to help.

○ 2

○ 1- Not done: The interviewer fails to inquire or acknowledge any impact of the illness on the patient's life or self-image.

PATIENT EDUCATION SKILLS

Patient Education/Provision of Information

○ 5- Well done: Summarizes key points of visit, clearly explains what they think is going on (cause of symptoms when appropriate). Explanations are helpful and give appropriate amount of information. Avoids medical jargon or immediately clarifies what is meant.

○ 4

○ 3- Partly done: Reviews some aspects of information covered during visit, some attempt to explain what they think is going on. May over or under explain. Minimal use of medical jargon.

○ 2

○ 1- Not done: Doesn't summarize key points of visit or explain what they think is going on. Explanations are either excessive or too minimal to be useful. Use of medical jargon.

© 2024 Gonzalez CM et al. *JAMA Network Open.*

Negotiates Plan and Provides Closure

- ○ 5- Well done: Asks for questions and checks for understanding. Negotiates and reaches agreement with patient on what is going to happen next.
- ○ 4
- ○ 3- Partly done: Asks for questions but may not check for understanding. Reviews what is going to happen next but does not fully elicit patient's agreement with plan.
- ○ 2
- ○ 1- Not done: Does not encourage patient to ask questions or check for understanding. Doesn't incorporate patient's preferences or inform patient of what is going to happen next.

© 2024 Gonzalez CM et al. *JAMA Network Open.*

Global Rating

<u>GLOBAL RATING: GUT CHECK! First answer is the best answer.</u>
Please provide a global rating of the physician you just saw.
Would you recommend this doctor to a friend/family member based on…

Inspires trust

- ○ Highly recommend
- ○ Recommend
- ○ Recommend with reservations
- ○ Not recommend

Confidence and medical knowledge

- ○ Highly recommend
- ○ Recommend
- ○ Recommend with reservations
- ○ Not recommend

Communication and interpersonal skills

- ○ Highly recommend
- ○ Recommend
- ○ Recommend with reservations
- ○ Not recommend

© 2024 Gonzalez CM et al. *JAMA Network Open.*

Explanation of treatment plan

- ○ Highly recommend
- ○ Recommend
- ○ Recommend with reservations
- ○ Not recommend

Based on your interaction with this physician *today only*, would you like to see this physician again?

- ○ I would very much like to see this physician again even if not convenient (worth following him/her to another practice).
- ○ I would like to see this physician again
- ○ I would be fine seeing this doctor again if assigned to me in an urgent situation
- ○ Would not like to see this physician again

Patient-physician rapport

This physician cares for this patient

- ○ Strongly agree
- ○ Agree
- ○ Neither agree nor disagree
- ○ Disagree
- ○ Strongly disagree

© 2024 Gonzalez CM et al. *JAMA Network Open.*

This physician has a *great deal* of respect for this patient

- ○ Strongly agree
- ○ Agree
- ○ Neither agree nor disagree
- ○ Disagree
- ○ Strongly disagree

Please enter any comments you wish to share about your physician's performance in the box below.

____

____

____

____

© 2024 Gonzalez CM et al. *JAMA Network Open.*

Appendix 2: Components of Implicit Association Tests

Category (Race IAT)	Items
Good	Joy, Love, Peace, Wonderful, Pleasure, Excellent, Laughter, Happy
Bad	Angry, Terrible, Horrible, Nasty, Evil, Awful, Failure, Hurt
African American	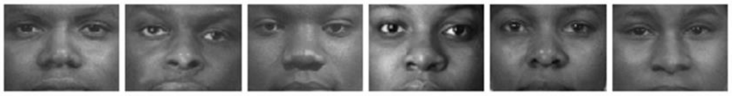
European American	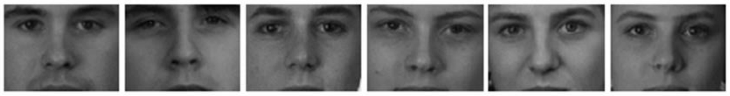

Category (Race-Medical Cooperativeness IAT)	Items
Compliant Patient	Willing, Cooperative, Compliant, Reliable, Adherent
Reluctant Patient	Reluctant, Doubting, Hesitant, Apathetic, Resistant
African American	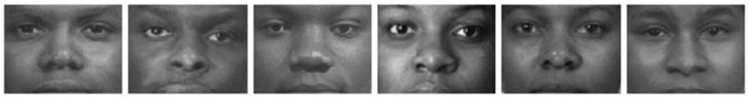
European American	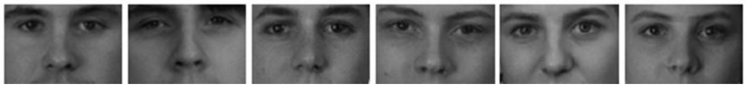

eAppendix 2. Components of Implicit Association Tests

Nosek, B. A., Smyth, F. L., Hansen, J. J., Devos, T., Lindner, N. M., Ratliff (Ranganath), K. A., Smith, C. T., Olson, K. R., Chugh, D., Greenwald, A. G., & Banaji, M. R. (2007). Pervasiveness and correlates of implicit attitudes and stereotypes. European Review of Social Psychology, 18, 36-88

© 2024 Gonzalez CM et al. *JAMA Network Open.*
