# Supplementary material for: Racial Implicit Bias and Communication Among Physicians in a Simulated Environment
Source: JAMA Netw Open. 2024 Mar 20;7(3):e242181. doi: 10.1001/jamanetworkopen.2024.2181 (PMC10955368; doi:10.1001/jamanetworkopen.2024.2181)
Supplement: Supplement 2. — Data Sharing Statement [file jamanetwopen-e242181-s002.pdf]

Data Sharing Statement

Gonzalez. Assessment of Racial Implicit Bias and Communication Among Physicians in a Simulated Environment. *JAMA Netw Open*. Published March 20, 2024. doi:10.1001/jamanetworkopen.2024.2181

Data
Data available: Yes
Data types: Deidentified participant data, Data dictionary
How to access data: Data requests can be sent to the corresponding author at Cristina.Gonzalez@nyulangone.org
When available: With publication

Supporting Documents
Document types: None

Additional Information
Who can access the data: Anyone requesting the data
Types of analyses: for the purposes of intervention design/evaluation (if focused on addressing implicit bias and/or simulation to improve patient outcomes
Mechanisms of data availability: With a signed data access agreement
Any additional restrictions: N/A
